# Supplementary material for: Remote detection of radioactive material using high-power pulsed electromagnetic radiation
Source: Nat Commun. 2017 May 9;8:15394. doi: 10.1038/ncomms15394 (PMC5436141; doi:10.1038/ncomms15394)
Supplement: Supplementary Information — Supplementary Figures, Supplementary Table, Supplementary Notes and Supplementary References. [file ncomms15394-s1.pdf]

Supplementary Information

Supplementary Figures

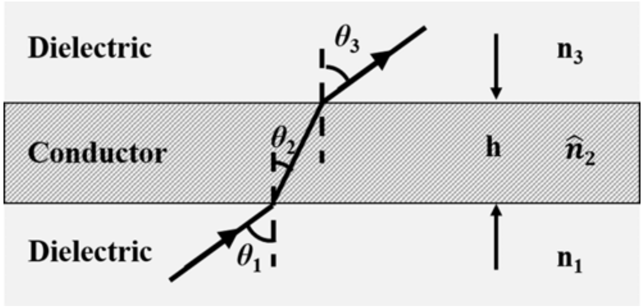

Supplementary Figure 1. **Absorbing material placed between two dielectric media**
The incident electromagnetic wave propagates in stratified dielectric media. Here, n_1 and n_3 represent the refractive index of the dielectric media, and $\hat{\boldsymbol{n}}_2$ represents the real part of the refractive index in the conductor. The thickness of the conductor is h. The electromagnetic wave propagation is represented as a black arrow. θ_1, θ_2, and θ_3 represent the incident angles of the wave at each media.

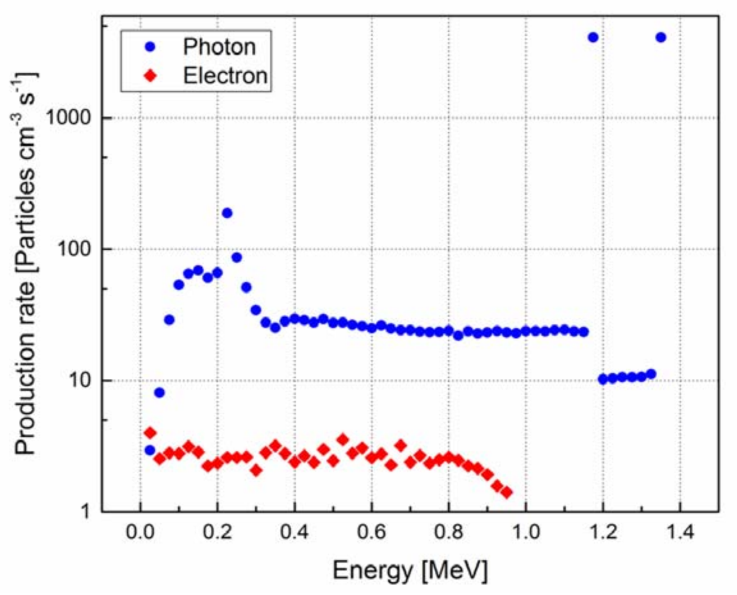

Supplementary Figure 2. **Energy spectrum of photons and electrons**
The production rate of high-energy photons and electrons owing to 0.64 mCi ^{60}Co located 20 cm away as function of energy as calculated using Monte Carlo N-Particle eXtended (MCNPX ver. 2.50) code.

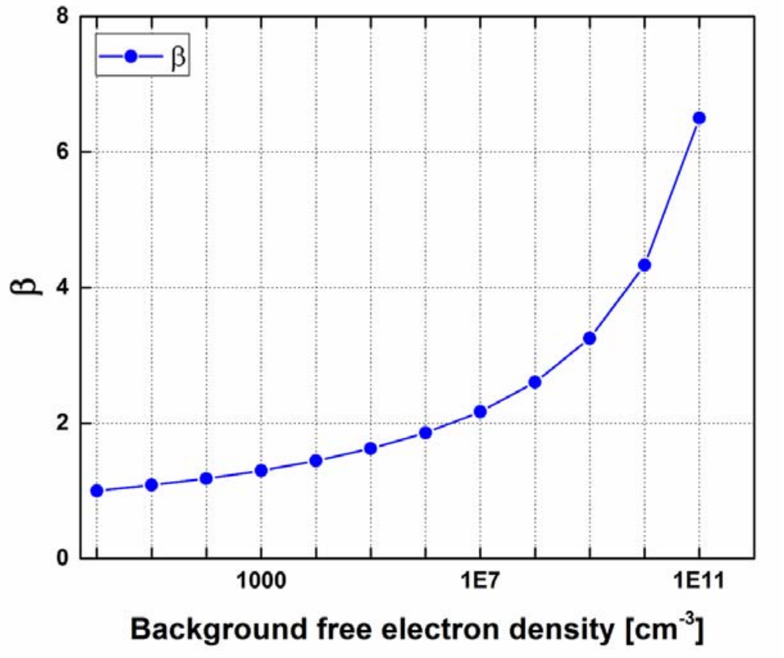

Supplementary Figure 3. **Electric field reduction factor vs free electron density**
Electric field reduction factor, β, as function of background free electron density as calculated using Supplementary Equation (33).

Supplementary Table 1. **Calculation of plasma densities based on the transmittance**

The plasma densities were calculated using transmittance of the attenuated RF pulse under different condition.

Pressure (gas)	Transmittance	Plasma density
760 Torr (air)	0.19	6.44×10^{13} cm^{-3}
760 Torr (Ar)	0.20	6.23×10^{13} cm^{-3}
60 Torr (air)	0.22	5.87×10^{13} cm^{-3}

Supplementary Note 1: Radiation dose rate calculation

Here we describe the calculation of the radiation dose rate (*D*):

$$D\,(\mathrm{Sv/h}) = \frac{\Gamma \times S}{d^2}, \qquad (1)$$

where $\Gamma = 1.32\left(\frac{R \cdot \mathrm{m}^2}{\mathrm{Ci} \cdot \mathrm{h}}\right)$ is the specific gamma-ray constant for ^{60}Co (*R* = 0.01 Sieverts (Sv)), *S* is the radioactivity (Ci), and *d* is the distance from the source (m). For example, the radiation dose rate of 1 mCi of pure ^{60}Co can be 1 μSv h^{-1} at 3.5 m away from the source.

The 0.64 mCi ^{60}Co source used in our experiment is equivalent to 0.5 μg of pure ^{60}Co (1 Ci = 3.7×10^{10} Bq, 2.8×10^{9} Bq = 60 μg). From the detectable mass equation (equation (3) in the manuscript), the available amount of ^{60}Co was determined to be approximately 2.4 mg at 1.2 m. This value was converted to 3.03 Ci, yielding *D* = 27.7 mSv h^{-1}.

Supplementary Note 2: Plasma density estimation

The plasma density in the main text was calculated based on the transmission of the RF wave. As shown in Fig. 2 in the manuscript, the incident RF wave was not completely attenuated, and small amounts of the RF signal were detected in air at 60 Torr, air at 760 Torr, and Ar at 760 Torr. The transmitted power was measured for the plasma density calculations. The plasma density was derived from a model of absorbing film between two dielectric media.[1]

As shown in Supplementary Fig. 1, the electromagnetic waves were assumed to propagate in stratified dielectric media. The complex refractive index of medium 2 is given by

$$\hat{n}_2 = n_2(1 + i\kappa_2), \tag{2}$$

where $\hat{n}_2$ is the real part and κ_2 is the imaginary part. Hence, the dielectric constant of a plasma can be simply defined as

$$\varepsilon = 1 - \frac{\omega_\mathrm{p}{}^2}{\omega^2 + i\omega\nu_\mathrm{eff}}, \tag{3}$$

where ω_p is the plasma frequency, ω is the angular frequency of the incident wave, and ν_eff is the effective collision frequency of the electrons.[2] The initial plasma density was assumed to be 1 cm^{-3} and to grow exponentially with time. Thus, the real and imaginary parts of the refractive index can be written as

$$n_2 = \sqrt{\frac{(\varepsilon)_\mathrm{re}}{2} + \sqrt{\left(\frac{(\varepsilon)_\mathrm{re}}{2}\right)^2 + \left(\frac{(\varepsilon)_\mathrm{im}}{2}\right)^2}} \tag{4}$$

and

$$\kappa_2 = \sqrt{-\frac{(\varepsilon)_\mathrm{re}}{2} + \sqrt{\left(\frac{(\varepsilon)_\mathrm{re}}{2}\right)^2 + \left(\frac{(\varepsilon)_\mathrm{im}}{2}\right)^2}}\ . \tag{5}$$

For convenience, the following expression was used in the calculations:

$$\hat{n}_2 \cos\theta_2 = u_2 + iv_2, \tag{6}$$

where u_2 and v_2 are real. Upon squaring Supplementary Equation (6) and substituting using Snell's law, $\hat{n}_2 \sin\theta_2 = n_1 \sin\theta_1$,

$$\left(u_2 + i\upsilon_2\right)^2 = \left(\hat{n}_2\right)^2 - \left(n_1 \sin\theta_1\right)^2 . \tag{7}$$

Supplementary Equation (7) can be separated into real and imaginary parts as follows:

$$\begin{aligned} u_2^{\ 2} - \upsilon_2^{\ 2} &= n_2^{\ 2}\left(1 - \kappa_2^{\ 2}\right) - n_1^{\ 2}\sin^2\theta_1 \\ u_2\upsilon_2 &= n_2^{\ 2}\kappa_2 \end{aligned} . \tag{8}$$

For the TE wave, the reflection and transmission coefficient at the first interface are given by

$$r_{12} = \rho_{12} e^{i\phi_{12}} = \frac{n_1\cos\theta_1 - (u_2 + i\upsilon_2)}{n_1\cos\theta_1 + (u_2 + i\upsilon_2)}, \tag{9}$$

$$\rho_{12}^{\ 2} = \frac{\left(n_1\cos\theta_1 - u_2\right)^2 + \upsilon_2^{\ 2}}{(n_1\cos\theta_1 + u_2)^2 + \upsilon_2^{\ 2}}, \quad \tan\phi_{12} = \frac{2\upsilon_2 n_1\cos\theta_1}{u_2^{\ 2} + \upsilon_2^{\ 2} - n_1^{\ 2}\cos^2\theta_1}, \tag{10}$$

$$t_{12} = \tau_{12} e^{i\chi_{12}} = \frac{2n_1\cos\theta_1}{n_1\cos\theta_1 + (u_2 + i\upsilon_2)}, \tag{11}$$

$$\tau_{12}^{\ 2} = \frac{\left(2n_1\cos\theta_1\right)^2}{(n_1\cos\theta_1 + u_2)^2 + \upsilon_2^{\ 2}}, \text{ and } \tan\chi_{12} = -\frac{\upsilon_2}{n_1\cos\theta_1 + u_2} \tag{12}$$

where ρ_{12} and τ_{12} are amplitudes and ϕ_{12} and χ_{12} are phase changes. Similarly, at the second interface, the amplitudes (ρ_{23} and τ_{23}) and phase changes (ϕ_{23} and χ_{23}) for the reflection and transmission coefficients are given by

$$\rho_{23}^{\ 2} = \frac{\left(n_3\cos\theta_3 - u_2\right)^2 + \upsilon_2^{\ 2}}{(n_3\cos\theta_3 + u_2)^2 + \upsilon_2^{\ 2}}, \quad \tan\phi_{23} = \frac{2\upsilon_2 n_3\cos\theta_3}{u_2^{\ 2} + \upsilon_2^{\ 2} - n_3^{\ 2}\cos^2\theta_3}, \tag{13}$$

$$\tau_{23}^{\ 2} = \frac{4\left(u_2^{\ 2} + \upsilon_2^{\ 2}\right)}{(n_3\cos\theta_3 + u_2)^2 + \upsilon_2^{\ 2}}, \text{ and } \tan\chi_{23} = \frac{\upsilon_2 n_3\cos\theta_3}{u_2^{\ 2} + \upsilon_2^{\ 2} + u_2 n_3\cos\theta_3}. \tag{14}$$

Upon combining Supplementary Equations (9)–(14), the reflectance and transmittance can be expressed as

$$R = |r|^2 = \frac{\rho_{12}^{\ 2} e^{2\upsilon_2\eta} + \rho_{23}^{\ 2} e^{-2\upsilon_2\eta} + 2\rho_{12}\rho_{23}\cos\left[\phi_{23} - \phi_{12} + 2u_2\eta\right]}{e^{2\upsilon_2\eta} + \rho_{12}^{\ 2}\rho_{23}^{\ 2} e^{-2\upsilon_2\eta} + 2\rho_{12}\rho_{23}\cos\left[\phi_{12} + \phi_{23} + 2u_2\eta\right]} \tag{15}$$

and

$$
\begin{aligned}
T &= \frac{n_3 \cos\theta_3}{n_1 \cos\theta_1} |t|^2 \\
&= \frac{n_3 \cos\theta_3}{n_1 \cos\theta_1} \cdot \frac{{\tau_{12}}^2 {\tau_{23}}^2 e^{-2 v_2 \eta}}{1 + {\rho_{12}}^2 {\rho_{23}}^2 e^{-4 v_2 \eta} + 2\rho_{12}\rho_{23} e^{-2 v_2 \eta} \cos\left[\phi_{12} + \phi_{23} + 2u_2\eta\right]}.
\end{aligned} \tag{16}
$$

Here, $\eta = 2\pi h/\lambda_0$, where h is the thickness of the conductor and λ_0 is the wavelength in a vacuum. With the calibrated RF detector, we could estimate the plasma density based on the transmittance values measured during the experiment. (See Supplementary Table 1.)

Supplementary Note 3: Derivation of the probability of no breakdown

We introduce the theoretical formative delay time derived from the electron continuity equation. The number density of free electrons at time t is given by

$$n(t) = n_{\mathrm{i}} \exp[\nu t]. \tag{17}$$

Here, n_{i} is the initial electron number density, and $\nu = \nu_{\mathrm{i}} - \nu_{\mathrm{a}} - \nu_{\mathrm{d}}$ is the net ionization rate in terms of ν_{i}, ν_{a}, and ν_{d}, which represent the ionization, attachment, and diffusion frequencies, respectively. For monatomic gases and inelastic collisions between electrons, the ionization frequency was derived by Raizer *et al.* as follows:[3]

$$\nu_{\mathrm{i}} = a^2 \times \nu_{\mathrm{E}} \times \alpha \times \beta, \tag{18}$$

$$\alpha = 2 \times a \times \exp\left(-\frac{a-1}{a} \times \sqrt{\frac{6\nu^*}{\nu_{\mathrm{E}}}} \right), \tag{19}$$

and

$$\nu_{\mathrm{E}} = 1.75 \times 10^{15} \times \left(\frac{E_{\mathrm{rms}}^2}{\omega^2 + \nu_{\mathrm{m}}^2} \right) \times \frac{\nu_{\mathrm{m}}}{I_1}, \tag{20}$$

where α = 1.2 is for monatomic gases, β = 0.2 is for breakdown in a constant field at a high and microwave frequency, $\nu^* = 2.6\times10^8 \times p$ (Torr s^{-1}) is the atomic excitation frequency for argon (Ar) gas, $\nu_{\mathrm{m}} = 7\times10^9\times p$ (Torr s^{-1}) is the rate of collisions between electrons and Ar particles, p is the chamber pressure, E_{rms} is the root-mean-square electric field amplitude calculated based on the incident gyrotron beam, and $\omega = 2\pi f$ (rad s^{-1}) is the angular frequency of the RF beam. The diffusion frequency is given by

$$\nu_{\mathrm{d}} = \frac{D}{\Lambda^2} = \frac{5.8\times10^{14}\times I^*}{\nu_{\mathrm{m}}\times\Lambda^2}, \tag{21}$$

where D is the diffusion rate, I^* = 11.5 eV, $\Lambda = \omega_0/\pi$ is the characteristic diffusion length, and $\omega_0 \approx 5$ mm is the beam waist.

The probability of an avalanche reaching a size of N electrons per volume based on Supplementary Equation (17) is given by the expression[4,5]

$$P(N) = \frac{1}{\bar{n}} \exp\left(-\frac{N}{\bar{n}} \right), \tag{22}$$

where $\bar{n}$ (cm^{-3}) is the average value of N when n_{i} = 1 cm^{-3} in Supplementary Equation (17) and is given by

$$\bar{n} = \exp\left[\int_0^t \left(\nu_\mathrm{i}(t') - \nu_\mathrm{d}\right) dt'\right]. \tag{23}$$

Upon combining Supplementary Equations (22) and (23), it becomes evident that, when the electron density approaches the critical density ($n_\mathrm{cr} \approx 10^{14}$ cm^{-3}), the plasma frequency is the same as the angular frequency ($f \approx 95$ GHz). Then, the probability of no breakdown can be expressed as

$$P_1\left(N < n_\mathrm{cr}, t\right) = \int_0^{n_\mathrm{cr}} P(N) dN = 1 - \exp\left(-\frac{n_\mathrm{cr}}{\bar{n}}\right). \tag{24}$$

The theoretical breakdown formation time, defined in the manuscript, is a function of the pressure and amplitude of the incident electric field.[3] In the absence of an external radioactive source, the plasma avalanche occurs with a random delay time, which is referred to as the statistical delay time. The delay time decreases sharply in the presence of radioactivity due to the increase in the average free electron density.[4,6]

The statistical delay time in the case where a radioactive material is present is calculated to fit the experimental results. This delay time indicates the period before the appearance of an initial electron to initiate the avalanche in the breakdown-prone volume. The Poisson distribution generated by the seeding source is given by[4,7]

$$P_2(n) = \frac{1}{n!}\left(S\Delta t\right)^n \exp\left(-S\Delta t\right), \tag{25}$$

where S is the average rate of electron generation by the seeding source. We assume that the S term is independent of the inner pressure of the chamber owing to the creation of free electrons by the radioactive isotope. The probability of finding zero electrons (n=0) per volume up to time t is

$$P_2\left(n = 0, t\right) = \exp\left(-St\right). \tag{26}$$

The source term, S=6 μs^{-1}, is empirically dependent on the background ionization rate owing to gamma-rays.

Therefore, the total delay time for plasma breakdown, described as the survival rate for a given pulse length t, is written as

$$P = P_1 + P_2. \tag{27}$$

Supplementary Note 4: Analysis of required electric field with radioactive material

Breakdown occurs when the electron density reaches the critical plasma density, which is defined as $n_{\text{cr}} = \frac{\omega^2 m \varepsilon_0}{e^2}$.

Therefore, the delay time for the occurrence of breakdown can be obtained under the condition

$$n_{\text{cr}} \le n_0 e^{\nu_{\text{eff,i}} \cdot \tau} \tag{28}$$

$$\text{or,} \quad \nu_{\text{eff,i}} \ge \frac{1}{\tau} \ln\left(\frac{n_{\text{cr}}}{n_0}\right). \tag{29}$$

The effective ionization rate, $\nu_{\text{eff,i}}$, depends on the amplitude of the RF field, E_0. Therefore, one can express the functional formula for the ionization rate as

$$\nu_{\text{eff,i}}\left(E_0\right) = \nu_{\text{am}} Y\left(\frac{E_0}{E_{\text{cr}}}\right), \tag{30}$$

where E_{cr} is the critical field for inducing breakdown, and ν_{am} is the typical dissociative attachment. For $E_0 = E_{\text{cr}}$, $Y(1) = 1$, which means that the ionization rate is equal to the rate of attachment to the molecules.[8] If the amplitude of the applied RF field is significantly greater than the critical field amplitude, then the ionization rate is higher than the attachment rate. This induces plasma breakdown.

From Supplementary Equation (30), one can get the inverse function of Y:

$$\frac{E_0}{E_{\text{cr}}} = Y^{-1}\left(\frac{\nu_{\text{eff,i}}\left(E_0\right)}{\nu_{\text{am}}}\right). \tag{31}$$

Because $Y = \frac{1}{\tau_{\text{p}} \nu_{\text{am}}} \ln\left(\frac{n_{\text{cr}}}{n_0}\right)$, the ratio of the threshold field to the critical field is

$$\frac{E_0}{E_{\text{cr}}} \propto \frac{1}{\tau_{\text{p}} \nu_{\text{am}}} \ln\left(\frac{n_{\text{cr}}}{n_0}\right). \tag{32}$$

Therefore, the threshold field (E_0) is inversely proportional to the pulse length (τ_{p}); this indicates that a longer RF pulse results in a decrease in the threshold field amplitude for

plasma breakdown.[9] Further, E_0 depends on the logarithm of the ratio of the critical plasma density to the number density of the initial seed electrons.

Supplementary Equation (32) provides insights into the decrease in the RF field required for breakdown (E_0) when a radioactive material is present. We postulate that the increased conductivity in the breakdown-prone volume leads to a decrease in the amplitude of the electric field for breakdown. We express the electric field required for breakdown in terms of the field-reduction factor, β, attributable to the presence of a radioactive material.

$$\beta E_0 = E_{\mathrm{cr}}, \tag{33}$$

where $\frac{1}{\beta} = \ln\left(\frac{n_{\mathrm{cr}}}{n_0}\right) / \ln\left(\frac{n_{\mathrm{cr}}}{n_0^*}\right) = \ln\left(\frac{n_0^*}{n_0}\right)$. Here, n_0 is the seed electron number density when there is no radioactive material and n_0^* is the seed electron number density in the presence of radioactive material.

The average number and energy of the high-energy electrons are approximately 50 and 0.44 MeV, respectively, as shown in Supplementary Fig. 2. Therefore, we can calculate the number of secondary knock-on electrons produced by a single high-energy electron as follows:

$$\frac{0.44\ \mathrm{MeV}}{34\ \mathrm{eV}} = 12600. \tag{34}$$

The time for the collision of a high-energy electron with a molecule can be estimated to be

$$t_{\mathrm{coll}} = \frac{l}{v} = 1.6 \times 10^{-9}\ \mathrm{s}, \tag{35}$$

where l is the mean free path for the high-energy electron and v is the velocity of the electron. The mean free path for the high-energy electron can be calculated as

$$l = \frac{1}{\sigma n}, \tag{36}$$

where σ is the scattering cross-section and n is the density of an air molecule. The scattering cross-section of an electron with an energy of 0.44 MeV is approximately 10^{-17} cm^{-2},[10] and n is approximately 10^{19} cm^{-3} at T=300 K and 1 atm pressure. Therefore, the mean free path for the high-energy electron is approximately 100 μm and the collision time is approximately 4×10^{-13} s. The total time for the generation of 12600 secondary knock-on electrons is approximately 5×10^{-9} s.

Therefore, for a duration of 1 μs before the plasma breakdown is induced owing to the applied RF pulse, the number density of the total secondary knock-on electrons generated by 50 high-energy electrons is approximately 1.3×10^{8} cm^{-3}.

The dependency of the threshold electric field on the number of free electrons can be obtained from Supplementary Equation (33) and is shown in Supplementary Fig. 3.

Supplementary References

1. Born, M. & Wolf, E. *Principles of optics: electromagnetic theory of propagation, interference and diffraction of light*. (Elsevier, 1980).
2. Cook, A. M., Hummelt, J. S., Shapiro, M. A., & Temkin, R. J. Measurements of electron avalanche formation time in W-band microwave air breakdown. *Phys. of Plasmas* **18**, 080707 (2011).
3. Raizer, Y. P. & Allen, J. E. *Gas Discharge Physics*, Vol. 2 (Berlin, Springer, 1997).
4. Foster, J., Krompholz, H. & Neuber, A., Investigation of the delay time distribution of high power microwave surface flashover. *Phys. Plasm*. **18**, 013502 (2011).
5. Wijsman, R. A. Breakdown probability of a low pressure gas discharge. *Phys. Rev.* **75**, 833-838 (1949).
6. Krile, J. T. & Neuber, A. A. Modeling statistical variations in high power microwave breakdown. *Appl. Phys. Lett.* **98**, 211502 (2011).
7. Dorozhkina, D. *et al.* Investigations of time delays in microwave breakdown initiation. *Phys. Plasmas* **13**, 013506 (2006).
8. Gurevich, A., Borisov, N., & Milikh, G., *Physics of Microwave Discharges: artificially ionized regions in the atmosphere* (CRC Press, 1997).
9. Gould, L. & Roberts, L. W. Breakdown of Air at Microwave Frequencies. *J. Appl. Phys.* **27**, 1162 (1956).
10. Phelps, A. V. & Pitchford, L. C. Anisotropic scattering of electrons by N_2 and its effect on electron transport. *Phys. Rev. A* **31**, 2932-2949 (1985).
